# CardiO Cycle: a pilot feasibility study of in-bed cycling in critically ill patients post cardiac surgery

**DOI:** 10.1186/s40814-020-00760-5

**Published:** 2021-01-07

**Authors:** Anastasia N. L. Newman, Michelle E. Kho, Jocelyn E. Harris, Nasim Zamir, Ellen McDonald, Alison Fox-Robichaud, Patricia Solomon, for the Canadian Critical Care Trials Group

**Affiliations:** 1 School of Rehabilitation Science, McMaster University, Hamilton, Ontario Canada; 2 Hamilton General Hospital, Hamilton, Ontario Canada; 3 Physiotherapy Department, St. Joseph’s Healthcare Hamilton, Hamilton, Ontario Canada; 4 College of Medicine, University of Saskatchewan, Saskatoon, Saskatchewan Canada; 5 Department of Medicine, Division of Critical Care, McMaster University, Hamilton, Ontario Canada

**Keywords:** Intensive care unit, Critical care, Physiotherapy, Rehabilitation, Cardiac surgery

## Abstract

**Background:**

In-bed cycling is a novel modality for the initiation of early mobilization in the intensive care unit. No study has investigated its use in the critically ill, off-track post cardiac surgery population. Before conducting an effectiveness trial, feasibility data are needed. The aim of this study was to determine the feasibility of in-bed cycling in a population of off-track cardiac surgery patients.

**Methods:**

We conducted a prospective feasibility study in a 16-bed adult cardiac surgery intensive care unit in Ontario, Canada. Previously ambulatory adults (≥ 18 years) who were mechanically ventilated for ≥ 72 h were enrolled within 3 to 7 days post cardiac surgery. Twenty minutes of in-bed cycling was delivered by ICU physiotherapists 5 days/week. The primary outcome, feasibility, was the percent of patient-cycling sessions that occurred when cycling was appropriate. The secondary outcome was cycling safety, measured as cycling discontinuation due to predetermined adverse events.

**Results:**

We screened 2074 patients, 29 met eligibility criteria, and 23 (92%) consented. Patients were male (78.26%) with a median [IQR] age of 76 [11] years, underwent isolated coronary bypass (39.1%), and had a median EuroScore II of 5.4 [7.8]. The mean (SD) time post-surgery to start of cycling was 5.9 (1.4) days. Patients were cycled on 80.5% (136/169) of eligible days, with limited physiotherapy staffing accounting for 48.5% of the missed patient-cycling sessions. During 136 sessions of cycling, 3 adverse events occurred in 3 individual patients. The incidence of an adverse event was 2.2 per 100 patient-cycling sessions (95% CI 0.50, 6.4).

**Conclusions:**

In-bed cycling with critically ill cardiac surgery patients is feasible with adequate physiotherapy staffing and appears to be safe. Future studies are needed to determine the effectiveness of this intervention in a larger sample.

**Trial registration:**

This trial was registered with Clinicaltrials.gov (NCT02976415). Registered November 29, 2016.

**Supplementary Information:**

The online version contains supplementary material available at 10.1186/s40814-020-00760-5.

## Key messages regarding feasibility

- What uncertainties existed regarding the feasibility?

It was not known whether it was feasible to enroll a population of critically ill, off-track patients post cardiac surgery, how easy it would be to identify potential patients and gain consent, and whether they would tolerate this rehabilitation modality.

- What are the key feasibility findings?
  It is feasible to enroll critically ill, off-track patients post cardiac surgery although stringent inclusion criteria limited our sample size.
- What are the implications of the feasibility findings for the design of the main study?
  Future studies involving critically ill, off-track patients post cardiac surgery should consider broader, less strict inclusion criteria, including patients who are extubated, those who failed extubation, and those who were readmitted to the ICU from the rehabilitation ward.

## Background

The number of cardiac surgeries has decreased significantly over the past decade, precipitated primarily by improvements in the use of percutaneous coronary interventions (PCI). As a result of increasing PCI use, patients who qualify for cardiac surgery are older with more co-morbidities [1]. The development of a critical illness occurs in approximately 3% of this population [2]. These patients have prolonged requirements for ventilatory and hemodynamic support (so called off-track) and may have limited mobilization in the initial post-operative phase.

Early mobilization of patients receiving intensive care unit (ICU)-level care may reduce the iatrogenic effects of critical care [3, 4]. Early mobilization includes the application of various modes of physical activity, including range of motion exercises, functional mobility, and ambulation [5]. In-bed cycling using a bedside cycle ergometer is a modality to initiate early mobilization in critically ill patients. The safety and feasibility of in-bed cycling in the non-cardiac surgical ICU population, when initiated within the first 4 days of ICU admission, has been documented [6, 7]. Kho and colleagues initiated cycling within 3 days of ICU admission to 33 patients in a medical-surgical ICU and noted infrequent cycling termination and no device dislodgements [7]. Similarly, a single-center randomized controlled trial (RCT) of in-bed cycling plus standard physiotherapy treatment in mechanically ventilated medical-surgical ICU patients was associated with a low safety occurrence rate of 4% [8]. Eggmann and colleagues’ RCT of early endurance and resistance training using an in-bed cycle ergometer versus standard physiotherapy noted a 0.2% adverse event rate [9]. Both RCTs included patients post cardiac surgery in their samples [8, 9]. Unfortunately, no subgroup analyses were performed in either study. It is unknown if any of these safety events occurred in the post-cardiac surgery population.

The benefits of in-bed cycling in critically ill patients [6–8] are conflicting with more recent studies suggesting no difference to conventional therapy [9, 10]. The majority of studies include medical or general surgical populations; the cardiac surgery population has received comparatively less attention. It is typical for on-track patients to be extubated within 8 to 12 h after surgery and transferred to the ward on post-operative day 1. These patients are able to partake in functional mobility shortly after surgery and will be discharged home between 3 and 5 days post-operatively. The critically ill (off-track) cardiac surgery patients experience longer ICU stays, often have significant hemodynamic issues, and may not tolerate exercise. Saphenous vein graft site integrity during cycling has yet to be evaluated, and no previous studies enrolling patients post cardiac surgery have documented graft site integrity while cycling. The purposes of this study were to determine (1) the feasibility of conducting in-bed cycling in a cardiac surgery ICU, (2) the feasibility of meeting a priori enrollment targets, and (3) the safety of in-bed cycling with off-track cardiac surgery patients.

## Methods

Between August 28, 2017, and March 29, 2019, we conducted a single-center pilot, prospective, feasibility study in a 16-bed adult cardiac surgery ICU in Hamilton, Ontario, Canada. Our study was approved by the Hamilton Integrated Research Ethics Board (project number 1999) and was registered with Clinicaltrials.gov (NCT02976415). Inclusion criteria were (1) cardiac surgery patients ≥ 18 years old, (2) ICU stay for > 3 but < 7 days [7, 11], (3) mechanically ventilated for > 72 h, and (4) able to ambulate independently, with or without a gait aid, before hospital admission. We excluded patients who consistently met one or more of the following criteria during the first 7 days after surgery: (1) uncomplicated post-operative course with expected discharge to ward within 24 to 72 h (on-track), (2) new onset of uncontrolled atrial fibrillation with a rate > 130 beats/min (bpm), (3) temporary pacemaker insertion (transvenous pacemaker) or external pacing with no underlying rhythm, (4) previous lower extremity injury that prevents cycling, (5) open or unstable saphenous vein graft incision sites, (6) presence of an intra-aortic balloon pump (IABP) or femoral sheath, (7) use of more than 4 inotropes or vasopressors. A research coordinator screened admissions to the cardiac ICU and obtained written informed consent from each patient or their next of kin.

### Intervention

Cycling was performed using the RT300 Supine Cycle, a portable in-bed device which provides passive cycling, active cycling, or a combination of both with the patient in a supine or semi-recumbent position [12]. Throughout a single treatment session, the RT300 assisted with passive cycling, allowing patients the option to cycle actively, or to rotate between active and passive cycling depending on their physical abilities at the time of cycling. The amount of active versus passive cycling performed during each cycling session was patient controlled. Patients were enrolled for a maximum of 28 days or until they were either able to ambulate or discharged from the unit.

Critical care physiotherapists delivered 20 min of in-bed cycling, Monday to Friday, to patients as part of their post-operative care. We chose 20 min for consistency with previous cycling research that included patients’ post-cardiac surgery [8, 9] and moderate-intensity aerobic exercise recommendations for patients with cardiovascular disease [13]. All physiotherapists had received a multi-day training session using the in-bed ergometer and had experience using the device. The session protocol included a 30-s warm-up at a rate of 5 revolutions per minute (RPM) followed by 19 min of cycling at a rate of 10 RPM and a 30 s cool down at 5 RPM. Resistance was set at 0.6 Newton-meters (Nm) and remained constant for all cycling sessions. Pedaling rate was patient-directed. Patients were monitored by the physiotherapist and the bedside nurse. Physiotherapists provided verbal support to each participant as a means of encouraging active participation. Cycling was performed once per day. Heart rate, oxygen saturation, blood pressure, pulmonary artery pressure (if available), and respiratory rate were documented before cycling, at 5-min intervals throughout the 20-min session and immediately post-cycling. Routine physiotherapy, which included chest physiotherapy, passive and active range of motion, bed exercises, progressive mobility (dangling at edge of bed), transfer training to bedside chair, standing, and ambulation as appropriate, was delivered in addition to cycling. Cycling sessions and routine physiotherapy were performed in separate sessions for all enrolled participants. Table 1 outlines the daily cycling exemptions and cycling termination criteria that were utilized once a patient qualified and was enrolled in the study.

**Table 1:** Daily cycling exemption and cycling termination criteria

**Daily cycling exemptions**	
Use of 4 or more inotropes or vasopressors	
Any increased titration of inotropic medication in the past 2 h	
Active myocardial ischemia as confirmed by bedside 12-lead electrocardiogram (ECG)	
Mean arterial pressure (MAP) < 60 mmHg or > 110 mmHg	
Heart rate < 40 bpm or > 140 bpm within the past 2 h	
New onset of uncontrolled atrial fibrillation with a rate greater than 130 beats/min	
Persistent SpO_2_ less than 88%, or out of what is typical for the patient, within the last 2 h	
Use of neuromuscular blockade within the past 4 h	
Severe agitation, as measured by the Richmond Agitation and Sedation Scale (score > 2)	
Presence of an intra-aortic balloon pump or femoral sheath	
Insertion of a temporary pacemaker (transvenous pacemaker) or those who are paced externally with no underlying rhythm	
Unstable saphenous vein graft site(s)	
Change in goals of care to palliation	
Patient or proxy refusal	
Team perception that cycling is not appropriate despite lack of listed exemption criteria	
**Cycling termination criteria**	
Sustained decrease in oxygen saturation of < 88% for > 2 min despite attempts to improve oxygenation (increasing FiO_2_, tracheal suctioning)	
Patient or proxy refusal after 2 attempts at encouragement to continue	
Unplanned extubation or decannulation	
Dehiscence of saphenous vein graft incision site(s)	
Sudden onset of severe agitation (Richmond Agitation and Sedation Scale (score > 2))	
Concerns for new onset of cardiac ischemia as per continuous telemetry	
Sudden onset of cardiac arrhythmias (including bradycardia < 40 bpm, tachycardia > 140 bpm, atrial fibrillation, right or left bundle branch block, sustained ventricular tachycardia, ventricular fibrillation, bigeminy, trigeminy)	
Hypotension with a systolic blood pressure < 90 mmHg	
Accidental removal of any lines or tubes (i.e., chest tubes, Jackson-Pratt drain, arterial line)	

We recorded the route of oxygen delivery, mechanical ventilation settings, presence of dialysis (hemodialysis or continuous renal replacement therapy (CRRT)), agitation, and delirium as assessed by the Richmond Agitation-Sedation Scale (RASS) [14], Confusion Assessment Method for ICU (CAM-ICU) [15], as well as the use of specific critical care medications (i.e., vasopressors, inotropes, opiates, benzodiazepines, sedatives, and antiarrhythmics) during cycling sessions.

### Primary and secondary outcomes

The primary outcome was feasibility. The feasibility outcomes were (1) the ability to implement in-bed cycling into daily physiotherapy practice at least 80% of the time that patients had no cycling exemptions (Table 1; eligible cycling opportunities) and (2) enrollment of 30 patients or recruitment over the 19-month enrollment period, whichever was achieved first.

The main secondary outcome was cycling safety. We defined the following potential adverse events a priori: (1) sustained hypertension for ≥ 2 min (systolic blood pressure greater than 160 mmHg (or out of range for what is typical for the patient)), (2) sustained hypotension for ≥ 2 min (systolic blood pressure less than 90 mmHg (or out of range for what is typical for the patient)), (3) cardiorespiratory arrest, (4) oxygen desaturation less than 88% for ≥ 2 min, (5) removal of any lines or tubes, (6) cardiac arrhythmias (new onset of uncontrolled atrial fibrillation, bigeminy, trigeminy, junctional, or heart block rhythm), (7) saphenous vein graft incision site dehiscence confirmed by the ICU intensivist, and (8) sustained increased agitation for ≥ 2 min (RASS score > + 2) [14].

Other secondary outcomes included consent rate, hospital all-cause mortality, ICU length of stay, 28-day mortality, EuroScore II, New York Heart Association Functional Classification scores, handgrip strength, Functional Status Score for the Intensive Care Unit (FSS-ICU), 2-min walk test (2MWT), Clinical Frailty Scale, and the number of cycling sessions per patient.

### Sample size estimation

As no previous safety and feasibility study has been conducted in the cardiac surgery population, we based our enrollment rates on a previous safety and feasibility study of in-bed cycling in a similar-sized medical-surgical ICU [7]. The likelihood of developing a critical illness post cardiac surgery is approximately 3% [2]. With average cardiac surgical case numbers of 1850/year at the Hamilton General Hospital, we anticipated 4.6 potential patients per month (87 patients total). As this group of potential patients would also include those meeting our exclusion criteria (i.e., patients with a palliative trajectory and non-mechanically ventilated patients who require other critical care interventions), we sought to enroll 30 mechanically ventilated, off-track cardiac surgery patients or recruit for a period of 19-months, whichever came first. A sample size of 30 was deemed sufficient to provide insights into the pragmatisms of recruitment challenges, cycle delivery, and safety with this population.

### Analysis

Descriptive statistics were used to analyze participant demographics (e.g., age, sex) and baseline data (e.g., surgery, EuroScore II, saphenous vein incision site). The feasibility outcome was the percent of cycling sessions that occurred compared to the number of eligible cycling opportunities. The safety outcome was the percentage of cycling sessions terminated prematurely due to the development of one of the 8 a priori adverse events. We calculated means and standard deviations (SD) and medians and interquartile ranges [IQR] for normally and non-normally distributed data respectfully. Incidence of adverse events was reported as number of events per 100 patient-cycling sessions with 95% confidence intervals (CI) [16]. Analysis was performed using SPSS (IBM Corporation, 2017).

### Progression criteria

Progression criteria are defined as criteria that inform the decision to progress from a pilot study to a larger definitive trial [17]. Meeting these criteria suggests that a large-scale randomized trial would be viable [17]. We were guided by the work of Avery et al. and employed a traffic light system for specifying our progression criteria [18]. Using this system, green “Go” indicated that all criteria has been met and a future, larger scale randomized trial should proceed, yellow “Amend” indicated that some changes should be made to the larger trial, and red “Stop” indicated that the investigators should not move forward with a larger trial. For our enrollment feasibility, an enrollment of ≥ 50% of qualifying patients was selected [19]. Progression criteria for cycling feasibility on eligible cycling days of ≥ 70% were chosen [19].

## Results

### Enrollment feasibility results

**Fig. 1:**
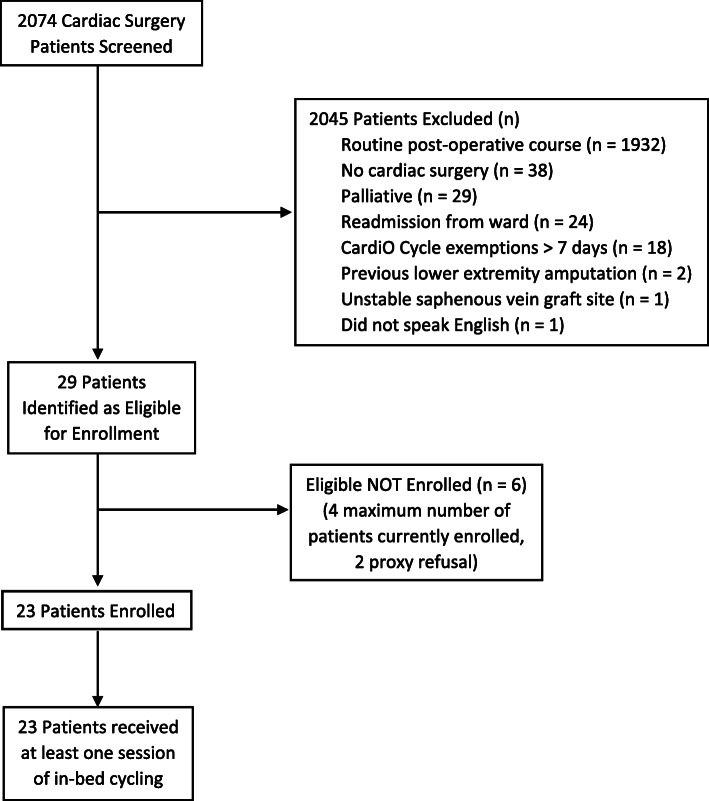
Patient flow diagram

We screened 2074 patients admitted to the ICU over the 19-month period from August 28, 2017, to March 29, 2019 (Fig. 1). We sought to enroll 30 patients. Twenty-nine patients met our inclusion criteria, 25 were offered enrollment, and 23 consented (23/25, 92% consent rate, Fig. 1). Four were not approached for consent due to the maximum capacity for physiotherapists to manage 2 concurrent patients at the time of study qualification. We fell short of our enrollment targets by 7 patients, achieving 76.7% of our recruitment goal. The majority of the 2045 patients who did not meet the inclusion criteria (1932/2045, 94.5%) were excluded from consideration due to their routine, uncomplicated post-operative course, and subsequent transfer from the ICU within 72 h post-surgery. Table 2 describes baseline demographic and clinical characteristics.

**Table 2:** Participant demographics and baseline information

Patient demographics	***N*** = 23 patients
**Age in years, median [IQR]**	76 [11]
**Males,** ***n*** **(%)**	18 (78.3%)
**Employment status,** ***n*** **(%)**	
**Retired**	16 (69.6%)
**Employed full-time**	2 (8.7%)
**Employed part-time**	1 (4.3%)
**Other/unknown**	4 (17.4%)
**Surgical procedure,** ***n*** **(%)**	
**Isolated coronary artery bypass graft (CABG)**	5 (21.7%)
**Single valve repair/replacement**	2 (8.7%)
**Other**^a^	16 (69.6%)
**History of previous cardiac surgery,** ***n*** **(%)**	6 (26.1%)
**Pre-operative New York Health Association (NYHA) functional classification**	
**NYHA I**	7 (30.4%)
**NYHA II**	3 (13%)
**NYHA II**	8 (34.8%)
**NYHA IV**	5 (21.7%)
**Pre-operative Canadian Classification Score (CCS)**	
**CCS I**	13 (56.5%)
**CCS II**	5 (21.7%)
**CCS III**	4 (17.4%)
**CCS IV**	1 (4.3%)
**Pre-operative Functional Independence Measure Scores, mean (SD)**^b^	125.5 (1.5)
**Pre-operative Functional Status Score–ICU, mean (SD)**	34.9 (0.3)
**Pre-operative Clinical Frailty Score**	
**Very fit (1)**	1 (4.3%)
**Well (2)**	9 (39.1%)
**Managing well (3)**	10 (43.5%)
**Vulnerable (4)**	2 (8.7%)
**Mildly frail (5)**	1 (4.3%)
**Moderately frail (6)**	0 (0%)
**Severely frail (7)**	0 (0%)
**Very severely frail (8)**	0 (0%)
**Terminally ill (9)**	0 (0%)
**Pre-operative EuroScore II, median [IQR]**	5.4 [7.8]
**Number of days mechanically ventilated (routine physiotherapy)**	98/150 (65.3%)
**Number of days mechanically ventilated (cycling)**	104/136 (76.5%)
**ICU length of stay (days), median [IQR]**	13 [21]
**Hospital length of stay (days), median [IQR]**	21 [29]
**ICU mortality,** ***n*** **(%)**	8 (34.8%)
**Hospital mortality,** ***n*** **(%)**	1 (4.3%)
**Discharge location of surviving participants from hospital,** ***n*** **(%)**	
**Home**	4 (28.6%)
**In-patient rehabilitation**	4 (28.6%)
**Repatriation to home hospital**	6 (42.9%)

^a^Maximum FIM Score 126.0

^b^Other surgeries include CABG plus valve replacement/repair, type A dissection repair, aortic root resection, double valve replacement/repair, Bentall’s procedure

The mean (SD) time from ICU admission post cardiac surgery to the initiation of cycling was 5.9 (1.4) days. Overall, our patients cycled a median [IQR] of 4 [2, 8] sessions which yielded 136 patient-cycling sessions and completed a mean (SD) of 113.7 (90.7) min of cycling per patient. Of the 136 patient-cycling sessions, 104 (76.5%) occurred while participants were mechanically ventilated via an endotracheal tube, 17 (12.5%) with a pulmonary artery catheter, and 14 (10.3%) during dialysis (8/136 CRRT (5.9%), 6/136 hemodialysis (4.4%)). Participants cycled during receipt of inotropes/vasopressors (35/136, 25.7%), benzodiazepines (22/136, 16.2%), and propofol (36/136, 26.5%). The median [IQR] RASS score pre-cycling was − 1 [1.75]. The 28-day mortality rate was 34.8%.

For patients enrolled in the study, 150 routine physiotherapy sessions were also conducted. These sessions occurred separately from cycling sessions. The most common routine interventions were airway clearance techniques, including manual percussions, vibrations, and tracheal suctioning (131/150, 87.3%), followed by passive range of motion exercises (115/150, 76.7%) and sitting at the edge of the bed (83/150, 55.3%).

Outcome measure results are available in Additional File 1.

### Cycling feasibility results

There were 169 eligible cycling days with cycling being delivered on 136 of those days (80.5%). Of the 33 days that cycling was not delivered, 16 (48.5%) were due to physiotherapy staffing shortages (e.g., uncovered sick and vacation days) and 6 (18.2%) occurred by participant discharge from ICU without advanced notification, preventing rescheduling of the cycling sessions (Table 3).

**Table 3:** Reasons for missed cycling sessions

Reasons for missed cycling	***N*** (%)
Staffing shortages	16 (48.5%)
Patient discharged from ICU	6 (18.2%)
Patient refusal	4 (12.1%)
Walking milestone achieved in ICU	4 (12.1%)
Daily CardiO Cycle exemption	2 (6.1%)
Bike incompatibility	1 (3.0%)
**Total**	**33**

### Cycling safety outcomes

Out of 136 patient-cycling sessions, 3 adverse events in 3 individual patients occurred with a calculated incidence of 2.2 per 100 patient-cycling sessions (95% CI 0.50, 6.4). Two patients developed hypotension while cycling. One patient became agitated with a RASS of greater than + 2. All 3 events lead to cycling termination with no further medical management required. Patients were able to cycle on subsequent days with no further events. The EuroScore II scores for these three patients were 1.58 (developed hypotension), 1.84 (developed agitation), and 3.71 (developed hypotension) respectively. These EuroScore II scores indicated low risk of post-operative mortality. We examined the saphenous vein graft incision sites before, during, and after cycling. No wound dehiscence was noted during our study period as determined by the intensivist on staff.

During routine physiotherapy, 5 adverse events occurred in 5 individual patients (5/150, 3.3%): 3 oxygen desaturations to < 88% for > 2 min despite attempts to improve oxygenation and 2 developments of cardiac arrhythmias (controlled atrial fibrillation) from previous normal sinus rhythm. No further medical management beyond terminating the physiotherapy intervention was required.

In the 136 patient-cycling sessions, participants cycled a total of 164.6 kilometers (km) with the median [IQR] distance cycled per participant of 5.07 [7.28] km and per session was 1.23 [0] km. The proportion of active versus passive cycling during a single patient-cycling session varied based on each patient’s status and available effort given at the time of cycling. Active cycling was noted in 45/136 patient-cycling sessions (33.1%). The minimum and maximum distances cycled during an individual session ranged from 0.49 to 2.04 km. The maximum total cumulative distance cycled by a single patient was 19.51 km. The majority (123/136, 90.4%) of the patient-cycling sessions were completed to the full protocol with the median [IQR] length of each patient-cycling session being 20.0 [1.3] min. The shortest cycling session was 7.9 min. The median [IQR] time to set up and complete cycling was 34 [5.7] min.

### Progression criteria results

For our enrollment feasibility, we enrolled 92% of our qualifying patients, which exceeded our target by 42%. Similarly, for our cycling feasibility, we were able to offer cycling on 80.5% of eligible cycling days, 10.5% above our target. This would correspond with the green traffic light color, as per Avery et al. [18].

## Discussion

Our study builds on the body of knowledge investigating the safety and feasibility of in-bed cycling with critically ill patients [6–9, 11, 20] and is the first to exclusively enroll off-track patients post complicated cardiac surgery. Our cycling protocol was safe, with only 3 adverse events occurring in 3 patients (incidence of 2.2 per 100 patient-cycling sessions), none of which required any further intervention other than cycling termination. This adverse event rate was similar to other ICU cycling studies (0.2–4%) [7, 8].

We obtained a high consent rate of 92% considering the acuity of our population. We screened 2074 patients; however, due to our narrow inclusion criteria, only 29 patients qualified. While our inclusion criteria allowed us to include patients with high acuity, it limited the enrollment of other patients who may have benefited from early in-bed cycling, such as those who required reintubation within 48 h of extubation, extubated patients who required prolonged hemodynamic support, and patients readmitted to ICU from the rehabilitation ward. While we exceeded our progression criteria with respect to recruitment of eligible patients, in consideration of future interventional trials in this population, the combination of our strict inclusion criteria and a high post-operative mortality rate would have limited the ability to assess the effectiveness of in-bed cycling on patients’ post-operative strength and functional abilities as well as their long-term functional outcomes. Future effectiveness trials of in-bed cycling in this population should consider broader inclusion criteria to prevent the exclusion of patients who may benefit from the early initiation of this modality, to ensure enrollment targets are met, and to increase the likelihood of assessing long-term functional outcomes.

Feasibility was dependent upon adequate physiotherapy staffing. “Adequate” staffing for critical care physiotherapists is not defined in the literature. However, staffing limitations have been identified as a common structural barrier that can have a significant impact on patient care [21–26]. In a 2015 survey of American critical care physiotherapists, half of the 550 respondents identified insufficient staffing as a barrier to the provision of physiotherapy [21]. Both Needham et al. [26] and Morris et al. [23] have noted the financial benefits of consistent physiotherapy staffing to hospitals via reduced lengths of stay with adequate staffing.

All patients were cycled on the day of their study enrollment with the mean (SD) time to initiation of cycling from ICU admission of 5.9 (1.4) days. This is more conservative than previous cycling literature [7, 9]. Time to cycling initiation from ICU admission was a median [IQR] of 1.98 [1.45] days and 3 [2] days in Eggmann et al. and Kho et al.’s studies respectively [7, 9]. In contrast, Burtin et al. initiated cycling on average 14 days after ICU admission [8]. Given that patients required at least 72 h of ICU admission before qualifying for study enrollment, cycling was performed within 3 days of study eligibility. Patients in our sample had a high median EuroScore II scores. Once patients were deemed medically stable, they were enrolled, and cycling was initiated. While the ideal timing to initiate early physical rehabilitation strategies with the critically ill is not known, it generally agreed upon that interventions should be delivered in the ICU [27]. Future randomized trials should consider the timing of exercise initiation as dictated by patient medical stability.

More than half our study patients (56.5%) had New York Heart Association Functional Classification scores greater than 3, signifying moderate to severe symptoms of congestive heart failure prior to surgery, and 78.2% of our sample scored between class I and II on the Canadian Cardiovascular Society Scores, indicating that symptoms of angina were present only during strenuous or moderate exertion. Over 80% of our sample were identified as either “Well” or “Managing Well” on the Clinical Frailty Scale [28, 29]. Despite the high operative risk, with a median EuroScore II of 5.4 [7.8], these patients were managing well at home and functionally independent prior to their cardiac surgery. Our mortality rate of 34% was similar to the 33.2% mortality rate in a 2018 population-based cohort study of long-term survival of post-cardiac surgery patients in Ontario [2]. Our sample spent a median of 13 [21] days in ICU, and 76.5% of all cycling sessions were conducted while the patient was mechanically ventilated (104/136). In contrast, patients who spent less than 2 days in ICU had a 1-year mortality rate of 2.1% [2]. These findings mirror the current trend of patients undergoing cardiac surgery who do not present as functionally compromised but who have multiple medical comorbidities which increases their operative risk [1, 2].

In comparison, patients identified as “on-track” post cardiac surgery tend to be younger in age, male, have intact left ventricle ejection fraction, no diagnosis of diabetes, and no previous history of either congestive heart failure or cardiac surgery [30]. The majority of these individuals will spend less than a day in ICU and will be discharged home within 5 days of surgery [2, 30]. As we continue to see an increase in the average age of the population undergoing cardiac surgery with underlying co-morbidities, this will impact the post-operative course for a large portion of patients with cardiovascular disease amenable to surgery. Identifying effective rehabilitation modalities to help mitigate the effects of prolonged critical care stays is essential and should be a focus of future physiotherapy research.

Our protocol of 20 min of in-bed cycling was consistent with previous cycling literature in which patients post cardiac surgery were enrolled [8, 9]. Eijsvogels et al. summarized the available evidence on the relationship between exercise volume and risk reductions in cardiovascular morbidity and mortality in patients identified as having cardiovascular disease, many who were post cardiac surgery [13]. Exercise volumes of 150 min/week, or 20 min/day, of moderate-intensity aerobic exercise were noted to reduce cardiovascular mortality. Considering patients were also receiving routine physiotherapy in addition to in-bed cycling, a 20-min protocol met previously published recommendations and seemed a feasible length of time for both our patients and physiotherapists.

Admission to ICU is not benign. The strength and functional impairments acquired during extended stays may remain well beyond hospital discharge. Physiotherapy may mitigate the effects of prolonged critical care stays [3, 31, 32]. However, the literature has been divisive with respect to the role of rehabilitation in treating the manifestations of weakness acquired in the ICU and whether the intensity of these interventions is enough to promote muscle strength gains and concomitant improvements in functional abilities. Schweickert et al. found that the initiation of early physiotherapy and occupational therapy in the ICU was associated with more patients achieving independent functional status at hospital discharge [3]. A 2018 systematic review of interventions to improve physical function of critically ill patients noted that early rehabilitation was associated with increased functional capacity, muscle strength, and improved walking distance at discharge [33]. In contrast, a recent RCT found no difference between the intervention group who received endurance training (with a cycle ergometer) and resistance training in combination with standard early mobilization as compared to the early mobilization group alone [9]. With the safety and feasibility documented, future research should focus on determining the effectiveness of this intervention in this population.

There are several presumed mechanisms on how muscles may respond to physical stress during the course of a critical illness, including the ability of mechanical signals to induce protein synthesis [34]. If muscles are able to respond to both passive stretch and mechanical stress with increased growth, the implementation of in-bed cycling can be justified as a means of mitigating the effects of prolonged critical care admissions. While only 33% of the cycling sessions in our study had periods of active cycling, the passive cycling may still contribute to promoting muscle strength gains by promoting muscle cell proliferation and growth [34]. This may justify the initiation of cycling while patients are intubated, sedated, and unable to actively contribute to cycling. Future studies need to investigate how best to physically challenge critically ill patients with consideration of long-term functional follow-up. Patient strength and functional outcomes, assessed using an outcome measure such as the FSS-ICU [35], post prolonged critical care stays in the cardiac surgical ICU are lacking and should be considered as a means of informing clinicians on best practice for ICU rehabilitation.

In this study, we demonstrated that in-bed cycling was potentially feasible and safe to implement with off-track critically ill patients post cardiac surgery. Future studies can build upon this safety and feasibility evaluation and previous effectiveness studies to determine how best to challenge critically ill patients post cardiac surgery in an effort to moderate the effects of prolonged ICU stays.

### Strength and limitations

This study is novel in that it is the first study to investigate the feasibility of in-bed cycling with a population of solely acutely critically ill, off-track cardiac surgery patients. We obtained a consent rate of 92% across our 19-month enrollment period. While our patients had high median pre-operative EuroScore II results, our findings suggest that in-bed cycling can be implemented with some of the most critically ill patients in the cardiac ICU.

Our study had limitations. We conducted a single-center study; thus, our results may not represent the broader critically ill cardiac surgery population. We did not meet our target due to our strict inclusion criteria. Future studies should incorporate broader inclusion criteria and target subgroups of the critically ill post cardiac surgery population missed by our strict criteria.

## Conclusions

In-bed cycling can be safely implemented in a population of off-track, critically ill patients post cardiac surgery and can be feasibly conducted by critical care physiotherapists with adequate staffing. The results of this single-center feasibility study can be the basis for future studies evaluating the effectiveness of this intervention in this high-risk group. The low adverse event rate can provide confidence to broaden the inclusion criteria to include and enroll patients with or without mechanical ventilation who continue to require critical care interventions in future research.

## Supplementary Information

**Additional file 1.** Outcome Measure Results.

## Data Availability

The datasets generated during the current study are available from the corresponding author on reasonable request.

## Abbreviations

2MWT: 2-min walk test
CABG: Coronary artery bypass graft
CAM-ICU: Confusion Assessment Method–Intensive Care Unit
CCS: Canadian classification score
CI: Confidence interval
CRRT: Continuous renal replacement therapy
ECG: Electrocardiogram
FSS-ICU: Functional Status Score for the Intensive Care Unit
IABP: Intra-aortic balloon pump
ICU: Intensive care unit
IQR: Interquartile range
MAP: Mean arterial pressure
NYHA: New York Health Association
PCI: Percutaneous coronary intervention
PFIT: Physical Function in Intensive Care Test
RASS: Richmond Agitation-Sedation Scale
RCT: Randomized controlled trial
SD: Standard deviation

## References

[1] Oakes G, Feindel C, Purdham D, Tu J, Wang J, Kingsbury K (2012). Report on adult cardiac surgery in ONTARIO. Cardiac care Network of Ontario in collaboration with Institute for Clinical Evaluative Sciences.

[2] McIsaac DI, McDonald B, Wong CA, van Walraven C (2018). Long-term survival and resource use in critically ill cardiac surgery patients: a population-based study. Can J Anaesth..

[3] Schweickert WD, Pohlman MC, Pohlman AS, Nigos C, Pawlik AJ, Esbrook CL (2009). Early physical and occupational therapy in mechanically ventilated, critically ill patients: a randomised controlled trial. Lancet..

[4] Azoulay E, Vincent JL, Angus DC, Arabi YM, Brochard L, Brett SJ (2017). Recovery after critical illness: putting the puzzle together-a consensus of 29. Crit Care..

[5] Hodgson CL, Berney S, Harrold M, Saxena M, Bellomo R (2013). Clinical review: early patient mobilization in the ICU. Crit Care..

[6] Kho ME, Martin RA, Toonstra AL, Zanni JM, Mantheiy EC, Nelliot A (2015). Feasibility and safety of in-bed cycling for physical rehabilitation in the intensive care unit. J Crit Care.

[7] Kho ME, Molloy AJ, Clarke FJ, Ajami D, McCaughan M, Obrovac K (2016). TryCYCLE: a prospective study of the safety and feasibility of early in-bed cycling in mechanically ventilated patients. PLoS One..

[8] Burtin C, Clerckx B, Robbeets C, Ferdinande P, Langer D, Troosters T (2009). Early exercise in critically ill patients enhances short-term functional recovery. Crit Care Med..

[9] Eggmann S, Verra ML, Luder G, Takala J, Jakob SM (2018). Effects of early, combined endurance and resistance training in mechanically ventilated, critically ill patients: a randomised controlled trial. PLoS One..

[10] Fossat G, Baudin F, Courtes L, Bobet S, Dupont A, Bretagnol A (2018). Effect of in-bed leg cycling and electrical stimulation of the quadriceps on global muscle strength in critically ill adults: a randomized clinical trial. JAMA..

[11] Kho ME, Molloy AJ, Clarke FJ, Reid JC, Herridge MS, Karachi T (2019). Multicentre pilot randomised clinical trial of early in-bed cycle ergometry with ventilated patients. BMJ Open Respir Res..

[12] Therapies R. RT 300 Supine Early Mobility Therapy System. n.d. Available from: https://www.restorative-therapies.com/rt300_supine_early_mobility_therapy_system?s=RT%20300. Accessed 10 Aug 2020.

[13] Eijsvogels TM, Molossi S, Lee DC, Emery MS, Thompson PD (2016). Exercise at the extremes: the amount of exercise to reduce cardiovascular events. J Am Coll Cardiol..

[14] Sessler CN, Gosnell MS, Grap MJ, Brophy GM, O’Neal PV, Keane KA (2002). The Richmond Agitation-Sedation Scale: validity and reliability in adult intensive care unit patients. Am J Respir Crit Care Med..

[15] Ely EW, Margolin R, Francis J, May L, Truman B, Dittus R (2001). Evaluation of delirium in critically ill patients: validation of the Confusion Assessment Method for the Intensive Care Unit (CAM-ICU). Crit Care Med..

[16] Thabane L, Ma J, Chu R, Cheng J, Ismaila A, Rios LP (2010). A tutorial on pilot studies: the what, why and how. BMC Med Res Methodol..

[17] Mbuagbaw L, Kosa SD, Lawson DO, Stalteri R, Olaiya OR, Alotaibi A (2019). The reporting of progression criteria in protocols of pilot trials designed to assess the feasibility of main trials is insufficient: a meta-epidemiological study. Pilot Feasibility Stud..

[18] Avery KN, Williamson PR, Gamble C, O’Connell Francischetto E, Metcalfe C, Davidson P (2017). Informing efficient randomised controlled trials: exploration of challenges in developing progression criteria for internal pilot studies. BMJ Open..

[19] Young HML, Goodliffe S, Madhani M, Phelps K, Regen E, Locke A (2019). Co-producing progression criteria for feasibility studies: a partnership between patient contributors, clinicians and researchers. Int J Environ Res Public Health..

[20] Kho ME, Molloy AJ, Clarke F, Herridge MS, Koo KK, Rudkowski J (2016). CYCLE pilot: a protocol for a pilot randomised study of early cycle ergometry versus routine physiotherapy in mechanically ventilated patients. BMJ Open..

[21] Malone D, Ridgeway K, Nordon-Craft A, Moss P, Schenkman M, Moss M (2015). Physical therapist practice in the intensive care unit: results of a national survey. Phys Ther..

[22] Dubb R, Nydahl P, Hermes C, Schwabbauer N, Toonstra A, Parker AM (2016). Barriers and strategies for early mobilization of patients in intensive care units. Ann Am Thorac Soc..

[23] Morris PE, Goad A, Thompson C, Taylor K, Harry B, Passmore L (2008). Early intensive care unit mobility therapy in the treatment of acute respiratory failure. Crit Care Med..

[24] Lord RK, Mayhew CR, Korupolu R, Mantheiy EC, Friedman MA, Palmer JB (2013). ICU early physical rehabilitation programs: financial modeling of cost savings. Crit Care Med..

[25] Leditschke IA, Green M, Irvine J, Bissett B, Mitchell IA (2012). What are the barriers to mobilizing intensive care patients?. Cardiopulm Phys Ther J..

[26] Needham DM, Korupolu R (2010). Rehabilitation quality improvement in an intensive care unit setting: implementation of a quality improvement model. Top Stroke Rehabil..

[27] Connolly B, O’Neill B, Salisbury L, Blackwood B (2016). Enhanced Recovery After Critical Illness Programme G. Physical rehabilitation interventions for adult patients during critical illness: an overview of systematic reviews. Thorax..

[28] Lee DH, Buth KJ, Martin BJ, Yip AM, Hirsch GM (2010). Frail patients are at increased risk for mortality and prolonged institutional care after cardiac surgery. Circulation..

[29] Rockwood K, Song X, MacKnight C, Bergman H, Hogan DB, McDowell I (2005). A global clinical measure of fitness and frailty in elderly people. CMAJ..

[30] Bainbridge D, Cheng D (2017). Current evidence on fast track cardiac recovery management. Eur Heart J Suppl..

[31] Adler J, Malone D (2012). Early mobilization in the intensive care unit: a systematic review. Cardiopulm Phys Ther J..

[32] Hodgson CL, Tipping CJ (2017). Physiotherapy management of intensive care unit-acquired weakness. J Physiother..

[33] Arias-Fernandez P, Romero-Martin M, Gomez-Salgado J, Fernandez-Garcia D (2018). Rehabilitation and early mobilization in the critical patient: systematic review. J Phys Ther Sci..

[34] Friedrich O, Reid MB, Van den Berghe G, Vanhorebeek I, Hermans G, Rich MM (2015). The sick and the weak: neuropathies/myopathies in the critically ill. Physiol Rev..

[35] Huang M, Chan KS, Zanni JM, Parry SM, Neto SG, Neto JA, et al. Functional Status Score for the ICU: an international clinimetric analysis of validity, responsiveness, and minimal important difference. Crit Care Med. 2016;44(12):e1155–64. 10.1097/CCM.0000000000001949 PMC5292321 27488220

