# Supplementary material for: CardiO Cycle: a pilot feasibility study of in-bed cycling in critically ill patients post cardiac surgery
Source: Pilot Feasibility Stud. 2021 Jan 7;7:13. doi: 10.1186/s40814-020-00760-5 (PMC7788703; doi:10.1186/s40814-020-00760-5)
Supplement: Supplementary file 1 — Additional file 1. Outcome Measure Results. [file 40814_2020_760_MOESM1_ESM.docx]

**Additional File 1: Outcome Measure Results**

| **Outcome Measure** | **ICU Awakening**  **(n = 18)** | **ICU Discharge**  **(n = 14)** | **Statistical Significance** |
| --- | --- | --- | --- |
| **FSS-ICU, median [IQR]** | 6.5 [4, 8.5] | 15.0 [9.8, 23.3] | p = 0.001 |
| **Handgrip Strength (lbs), median [IQR]** | Right: 3.7 [0.5, 18.8]  Left: 3.0 [0, 17.7] | Right: 20.2 [4.8, 25.8]  Left: 15.7 [3.8, 27.7] | p = 0.03  p = 0.04 |
| **2MWT (m)** | 0, 2^i^ | 27 [21.6, 32.6]^ii^ | N/A |

^i^ Results from 2 patients who completed the 2MWT as part of the awakening assessment

^ii^ Results from 7 patients who completed the 2MWT as part of the discharge assessment

**FSS-ICU = Functional Status Score for the Intensive Care Unit; ICU = Intensive Care Unit; IQR = Interquartile Range; 2MWT = Two Minute Walk Test**
